# Are Coiled-Coils of Dimeric Kinesins Unwound during Their Walking on Microtubule?

**DOI:** 10.1371/journal.pone.0036071

**Published:** 2012-04-27

**Authors:** Zhao-Wen Duan, Ping Xie, Wei Li, Peng-Ye Wang

**Affiliations:** Key Laboratory of Soft Matter Physics and Beijing National Laboratory for Condensed Matter Physics, Institute of Physics, Chinese Academy of Sciences, Beijing, China; Uni. of South Florida, United States of America

## Abstract

Dimeric kinesin motor proteins such as homodimeric kinesin-1, homodimeric Ncd and heterodimeric Kar3/Vik1are composed of two head domains which are connected together by a rod-shaped, coiled-coil stalk. Despite the extensive and intensive studies on structures, kinetics, dynamics and walking mechanism of the dimers, whether their coiled-coils are unwound or not during their walking on the microtubule is still an unclear issue. Here, we try to clarify this issue by using molecular dynamics simulations. Our simulation results showed that, for Ncd, a large change in potential of mean force is required to unwind the coiled-coil by only several pairs of residues. For both Ncd and kinesin-1, the force required to initiate the coiled-coil unwinding is larger than that required for unfolding of the single 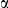-helix that forms the coiled-coil or is larger than that required to unwind the DNA duplex, which is higher than the unbinding force of the kinesin head from the microtubule in strong microtubule-binding states. Based on these results and the comparison of the sequence between the coiled-coil of Kar3/Vik1 and those of Ncd and kinesin-1, it was deduced that the coiled-coil of the Kar3/Vik1 should also be very stable. Thus, we concluded that the coiled-coils of kinesin-1, Ncd and Kar3/Vik1 are almost impossible to unwind during their walking on the microtubule.

## Introduction

Kinesin is one of the motor protein families that are involved in organelle transport, chromosome movement during cell division, and spindle formation by hauling cargoes over long distances along microtubule tracks [1], [2]. A conventional kinesin (kinesin-1) contains a light chain and a heavy chain which is composed of a ∼340 amino acid (AA) N-terminal motor domain which possesses ATPase and microtubule binding activities, a ∼485 AA 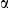-helical stalk, and a ∼92 AA C-terminal domain for cargo binding [3]. Typically, kinesin presents as a functional dimer where the stalk forms a rigid coiled-coil neck near the C-terminals of the heads [4]. Conventional kinesin-1 walks along microtubule toward the plus end in an asymmetric hand-over-hand manner [5], [6], [7], [8]. The ∼14 residue neck linker (NL) connecting the 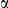-helical stalk and the heads was thought to play an important role in the kinesin-1 motility [9], [10], [11], [12]. The neck linker can undergo a large conformational change from an undocked state to a docked state upon ATP binding [13]. It was thought that the two long neck linkers allow both heads to bind the two successive binding sites along one protofilament of the microtubule simultaneously in rigor state [14], [15], [16], ensuring the processive walking.

Although most kinesins step toward the plus end of microtubule [17], kinesin-14 (K14), a subfamily, was observed to move toward the opposite direction [18], [19]. K14 distinguishes itself from other subfamilies not only in the walking direction along microtubule but also in the sequence [20]. Contrary to the conventional kinesin, K14 has an N-terminal tail domain and a C-terminal head domain [20]. The most well studied motor protein in K14 family is *Drosophila* Ncd. The crystal structure of homodimeric Ncd has shown that it has very short neck linkers (containing only three residues in each neck linker) and the distance between the two microtubule-binding sites in the motor domains is about 4.4 nm [21]. These structural properties and other experimental data indicate that Ncd is able to bind to the microtubule by only one of its two heads [22], [23], [24], [25]. Crystal structural studies of mutant Ncd dimer showed that there is an about 70 degree of rotation between its neck and the head which is binding to microtubule [26], [27]. The rotation which makes the neck point to the minus-end of the microtubule is thought to be the origin of minus-end walking of the protein [28], [29].

Unlike Ncd and many other members in K-14 family, the functional forms of Kar3, another protein in K-14 family, are heterodimers *in vivo* with either of two nonmotor proteins Vik1 or Cik1 [30], [31], [32], [33], [34], [35], although it can form homodimer *in vitro* [34]. Vik1 can localize Kar3 at the mitotic spindle poles, while Cik1 can promote accumulation of Kar3 along the length of the spindle in the absent of Vik1 [31], [33]. A recent study showed that both heads of the Kar3/Vik1 heterodimer can bind to microtubule simultaneously. Although the crystal structures of both Kar3 monomer and Vik1 monomer are available now, no crystal structure of Kar3/Vik1 dimer is available [36], [37]. The structure of Kar3 showed that its head domain is analogous to other kinesin heads, which possesses the ATPase activity, whereas Vik1 lacks the ATPase activity.

Despite the extensive and intensive studies, whether the coiled-coils of kinesin dimers are unwound or not during their walking along the microtubule is still an unclear issue [28], [38]. For example, it was assumed that the unwinding and over-winding of the coiled-coil induce the asymmetric hand-over-hand motion of kinesin-1 [6], while other models assumed that the coiled-coil is unnecessary to unwind and over-winding for the explanation of the asymmetric motion [6], [39]. More importantly, several models were proposed that the walking mechanism of Kar3/Vik1 heterodimer on the microtubule is relied on the unwinding of the coiled-coil [36], [38], [40]. The purpose of this work is to try to clarify this issue by using molecular dynamics (MD) simulations. Our simulation results showed that a large force or a large change of potential of mean force is required to unwind the coiled-coil of the kinesin dimers by only several pairs of residues, with the force being higher than the unbinding force of the kinesin head from microtubule. Thus, the unwinding of the coiled-coils of the kinesin dimers during their walking along the microtubule is almost impossible.

## Results

### Unwinding of the coiled-coil under the external potential

To study the unwinding dynamics of the coiled-coil, we fixed one head and exerted external potentials to the other head (see Materials and Methods), thus pulling apart the two heads. This somewhat resembles the real situation when kinesin walks along the microtubule. Since the neck linker of kinesin-1 has a long length (with 14 residues) and the two heads can be easily separated without the unwinding of its coiled-coil, it is too time-consuming to obtain the dimeric configuration with the unwound coiled-coil when we pull apart the two heads. Thus, we here only considered the case of Ncd because its neck linkers are much shorter than those of kinesin-1. There are 51 residues (Leu^296^ to Arg^346^) in the coiled-coil region in one monomer of the truncated dimeric Ncd (PDB 1CZ7) and the equilibrium distance between the two heads is about 4.38 nm [26]. Since a very large value of pulling speed (or loading rate) could produce different results for structural change from those in real situation, in our simulations we take the pulling speed to be as small as possible. We noticed that when the pulling speed was below 2
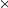
10^−3^ nm/ps, the obtained results for the structural change become insensitive to the pulling speed. Thus, in the simulation of the conformational trajectories which were used to calculate the change of potential of mean force, we took the pulling speed to be 2
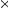
10^−4^ nm/ps that is 10-fold smaller than 2
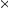
10^−3^ nm/ps.

**Figure 1:**
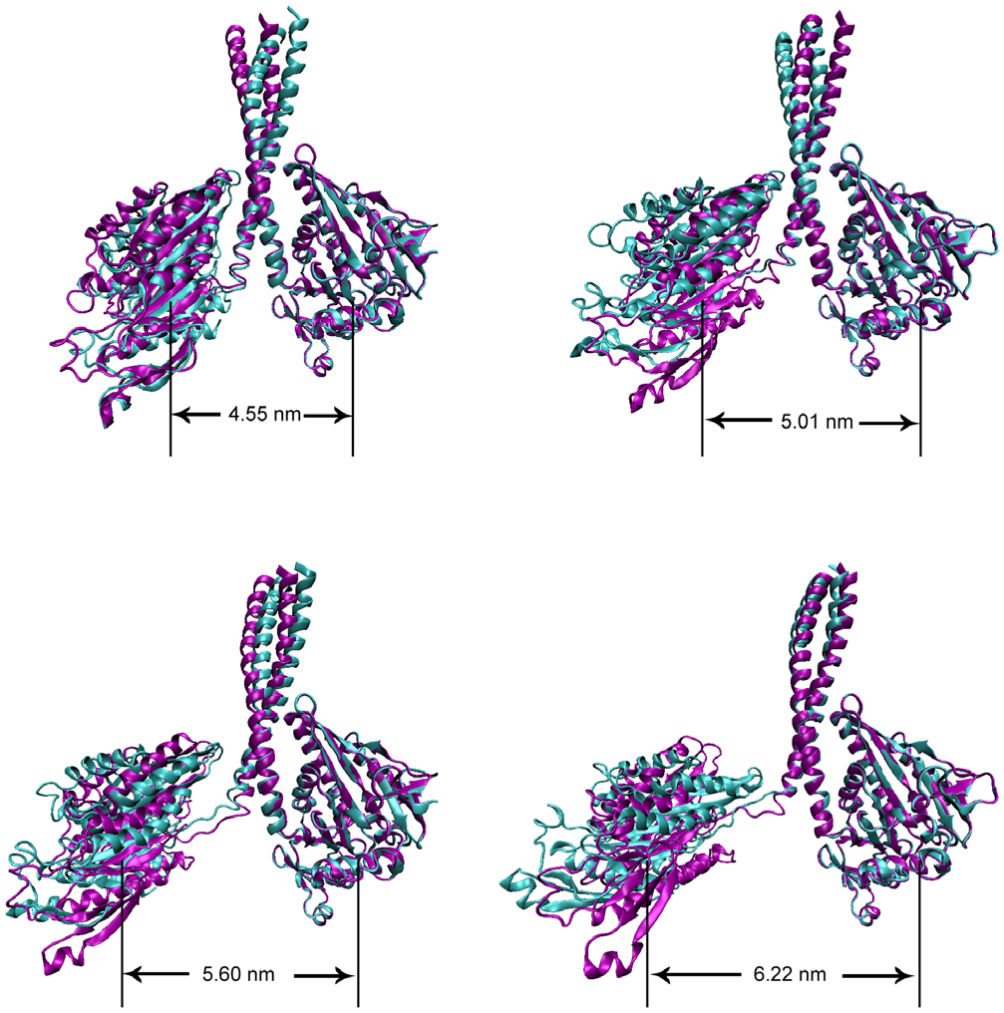
Snapshots of the pulling trajectories for Ncd dimer (Movie S1). The molecule in purple is from snapshots of trajectory under the pulling speed of 2
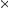
10^−3^ nm/ps while the molecule in cyan is from snapshots of trajectory under the pulling speed of 2
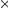
10^−4^ nm/ps. Both of the trajectories are under harmonic external potential and nucleotide-free state. The right heads of the dimer for both the pulling speeds are fixed and a harmonic potential is applied to the left head. Until *r_H_* = 4.55 nm no pair of residues in the coiled-coil region is unwound. From *r_H_* = 5.01 nm to the end, the coiled-coil is gradually unwound. The unwinding processes in both trajectories are almost identical.

Figure 1 shows some frames of the trajectories when the coiled-coil region of the nucleotide-free Ncd was unwound under a harmonic external field, where the Ncd molecule was rendered in purple under the pulling speed of 2
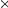
10^−3^ nm/ps and in cyan under the pulling speed of 2
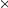
10^−4^ nm/ps (see Movie S1). As mentioned above, it is seen from Figure 1 that the processes under two pulling speeds were very similar. Before the initiation of coiled-coil unwinding, the whole molecule underwent slight deformation (Figure 1, *r*_H_ = 4.55 nm), giving the distance between the two heads being increased slightly from *r*_H_ = 4.38 nm to *r*_H_ = 4.55 nm. When the distance was gradually increased to a critical value, the coiled-coil was initiated to unwind (Figure 1, *r*_H_ = 5.01 nm). As the distance was further increased, more and more pairs of residues in the coiled-coil were unwound and, accompanying the unwinding, the α-helixes that form the coiled-coils were also unfolded (Figure 1, *r*_H_ = 5.60 nm and *r*_H_ = 6.22 nm). At the end of our pulling process (Figure 1, *r*_H_ = 6.22 nm) the coiled-coils were finally unwound by about 8 pairs of residues (His^339^ to Arg^346^).

To take into account the effect of the form of external force fields on the conformational change when coiled-coil was unwound, we replace the harmonic potential by a linear potential which can provide constant force to stretch the heads. The trajectories under the linear potential showed the similar pattern: accompanying the unwinding of coiled-coil the 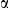-helix that forms the coiled-coil was also unfolded (see upper panel of Fig. S1). These results indicated that different forms of the external force field nearly have no effect on the way of coiled-coil unwinding. It is interesting to explore the way of coiled-coil unwinding under other external fields which is more relevant to the physiological case. However, our simulation software, GROMACS4, can only produce the above two forms of the external fields, which are also generally used in most of the pulling force experiments. To investigate whether the nucleotide states would affect the way of coiled-coil unwinding, we also run the simulations with ADP molecules bound to the heads of Ncd, with the results being shown in the lower panels of Fig. S1. The results indicated that the nucleotide binding states of Ncd also nearly have no effect on the way of coiled-coil unwinding. Taken together, it can be seen that under the simulation condition, the unwinding of coiled-coil would be accompanied with the unfolding of its helix, and the unwinding process is nearly independent of the form of external field and the nucleotide state of the molecule.

**Figure 2:**
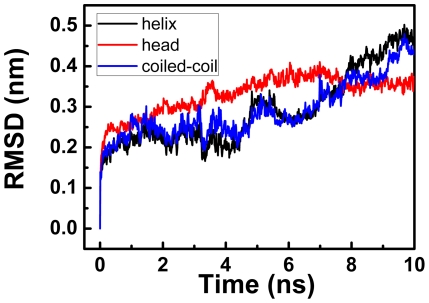
Root mean square deviations (RMSDs) of the coiled-coil region, the unfolded helix in coiled-coil and the heads of Ncd.

To quantitatively characterize the conformational change of the Ncd molecule, in Figure 2 we show the root mean square deviations (RMSDs) for the coiled-coil region, for the single unfolded α-helix and for the head domain under the pulling speed of 2
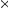
10^−4^ nm/ps, which were calculated from the trajectory shown in Figure 1. The RMSDs under the pulling speed of 2
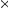
10^−3^ nm/ps were also similar to those in Figure 2 (not shown). It is noted here that, since the coiled-coil unwinding trajectories for different realizations are very similar, the RMSDs that are calculated from one specific trajectory would be not very different from those that are calculated from the average of trajectories with different realizations. As expected, the RMSDs of the coiled-coil region and the unfolded α-helix increase largely with the increase of the distance between the two heads. Moreover, their RMSD profiles are very similar, potentially indicating that the unwinding of coiled-coil was coupled with the unfolding of the helix (more discussion on the coupled unfolding will be presented later). From Figure 2 it is also seen that the RMSD of the head domain also increases largely, comparable to that of the coiled-coil region, but the conformational changes in head domain were not obvious (Figure 1 and Figure S1). This is explained as follows. The α-helix was also unfolded when the coiled-coil was unwound. The flexible, unfolded α-helix increases the extent to which the head can diffuse freely around its original position in solution, thus increasing largely the RMSD of the head domain.

### A large change in potential of mean force is required to unwind the coiled-coil by several pairs of residues

**Figure 3:**
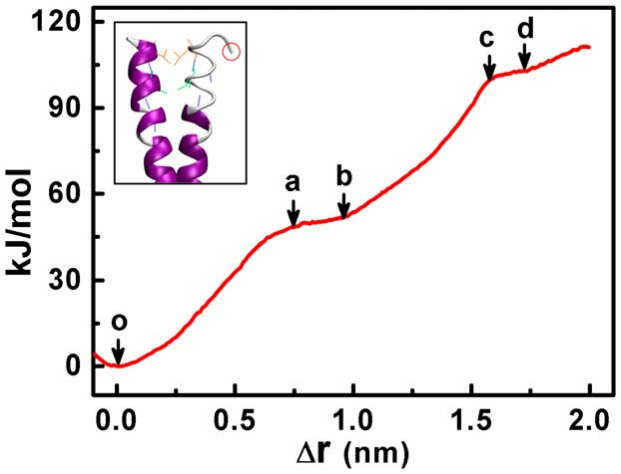
The potential of mean force change versus distance 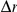
 **.** The distance 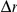 is defined as the positional change of residue Asn^348^ along the direction of pulling force, with 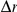 = 0 corresponding to position under no pulling force or energy minimum. Inset is the configuration of the coiled-coil under no pulling force, i.e., the configuration at point o. Residue Asn^348^ is indicated by the red circle.

From a series of molecular configurations during the pulling process under a pulling rate of 2
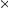
10^−4^ nm/ps (Figure 1), we calculated the change in potential of mean force resulting from the conformational change of the coiled-coil. For this purpose, we used the series of configurations of the coiled-coil region by cutting off the heads and fixing one helix of the coiled-coil. It should be noted that, since the large RMSD of the heads could also induce the increase of the potential of mean force, the change of the potential of mean force calculated here by considering only the coiled-coil should be smaller than that by considering both the coiled-coil and the heads. Here we denote 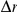 the positional change of residue Asn^348^ (indicated by red circle in the inset of Figure 3) along the direction of pulling force, with 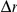 = 0 corresponding to position of the lowest potential of mean force (point o in Figure 3). Based on the umbrella sampling method, the results of the change in potential of mean force versus 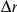 could be obtained (Figure 3).

**Figure 4:**
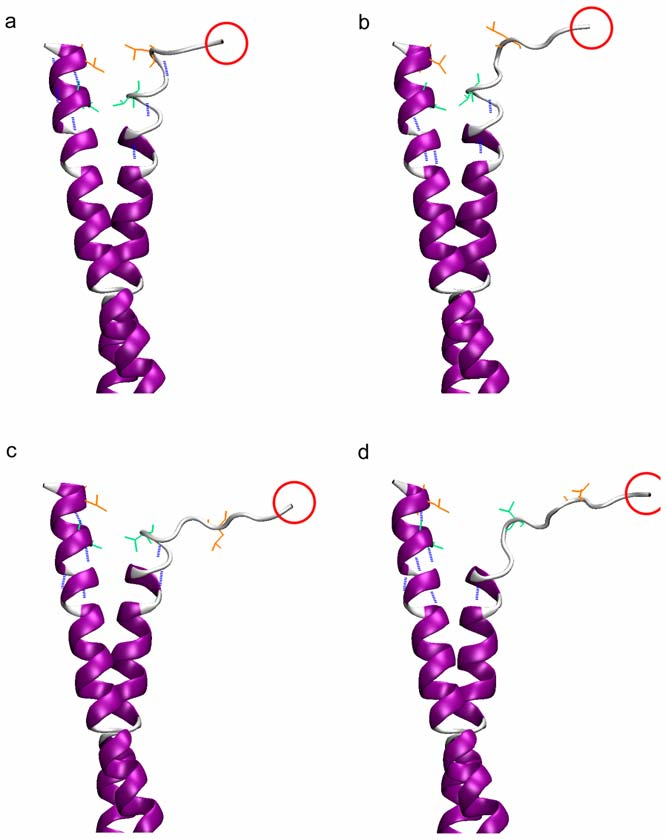
Configurations of coiled-coil. The configurations in (a), (b), (c) and (d) correspond to those at point a, b, c and d in Figure 3, respectively. The hydrophobic residues in orange are residues Leu345 and in green are residues Val342. Each pair of these residues are attracted via hydrophobic interaction. Hydrogen bonds in the helix are represented by blue dash lines. From Figure 4a to 4b, the pair of residues Leu^345^ was unwound and the unwound helix was unfolded via breaking one hydrogen bond (the blue dash line indicated by the arrow, Figure 4). From Figure 4c to 4d the pair of residues Val^342^ were unwound and the unwound helix was unfolded via breaking one hydrogen bond.

The increase process of the potential of mean force versus 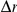 could be represented by two types of phases (Figure 3). One type is from point o to point a (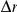
<0.75 nm) or from points b to point c (0.98 nm<
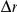
<1.55 nm), where the potential of mean force increases quickly with the increase of 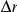 and the potential of mean force change in each phase is about 45 kJ/mol. From the configurations in the trajectory it is noted that the conformational changes in this type of phase involves only the deformations of coiled-coil without either unwinding of coiled-coil or unfolding of the helixes, i.e., no pair of coiled-coil residues was unwound and no unfolding of helixes occurred until 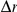 equals 0.75 nm (inset of Figure 3 and Figure 4a) or when 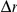 increases from 0.98 nm to 1.55 nm (Figure 4, b and c). The other type of phase is from point a to point b (0.75 nm<
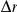
<0.98 nm) or from point c to point d (1.55 nm<
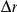
<1.74 nm), where the potential of mean force increases slowly with the increase of 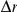. The small potential of mean force change in this type of phase corresponds to the unwinding of one pair of coiled-coil residues and the unfolding of the unwound helix (Figure 4a–4b and 4c–4d).

From Figure 3 it is seen that the total increase in potential of mean force of the coiled-coil in dimeric Ncd was about 111.3 kJ/mol when its coiled-coil was partially unwound and the neck linker residue were stretched by 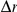 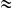
2 nm. If the two heads of Ncd bind simultaneously the two successive binding sites along one protofilament of the microtubule, based on the crystal structure of microtubules, 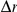 should reach about 3.8 nm and, thus, the potential of mean force increase should be much higher than the above value. This high increase in potential of mean force is impossible to be triggered by the thermal noise. Thus, only one head can bind to the microtubule during its moving on the microtubule [22], [23], [24], [25].

The crystal structure of Vik1 showed that it also has a short neck linker that contains only 3 residues (Gly^373^ to Met^375^) [36]. Although the head of Kar3 has been resolved [37], neither the structure of its neck linker nor that of its neck region is available now. However, since Kar3, like Ncd, is a kinesin-14 family protein, it is most possible that it also has a short neck linker that contains only 3 residues (see below). Thus, like homodimeric Ncd, without the unwinding of its coiled-coil, the two heads of heterodimeric Kar3/Vik1 is unable to simultaneously bind the two successive binding sites along one protofilament of the microtubule. Detailed discussions on the possibility of the coiled-coil unwinding and the walking mechanism of Kar3/Vik1 will be presented later.

### The force required to unwind the coiled-coil is larger than that to unfold the single 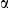-helix that forms the coiled-coil or is larger than that to unwind DNA duplex

**Figure 5:**
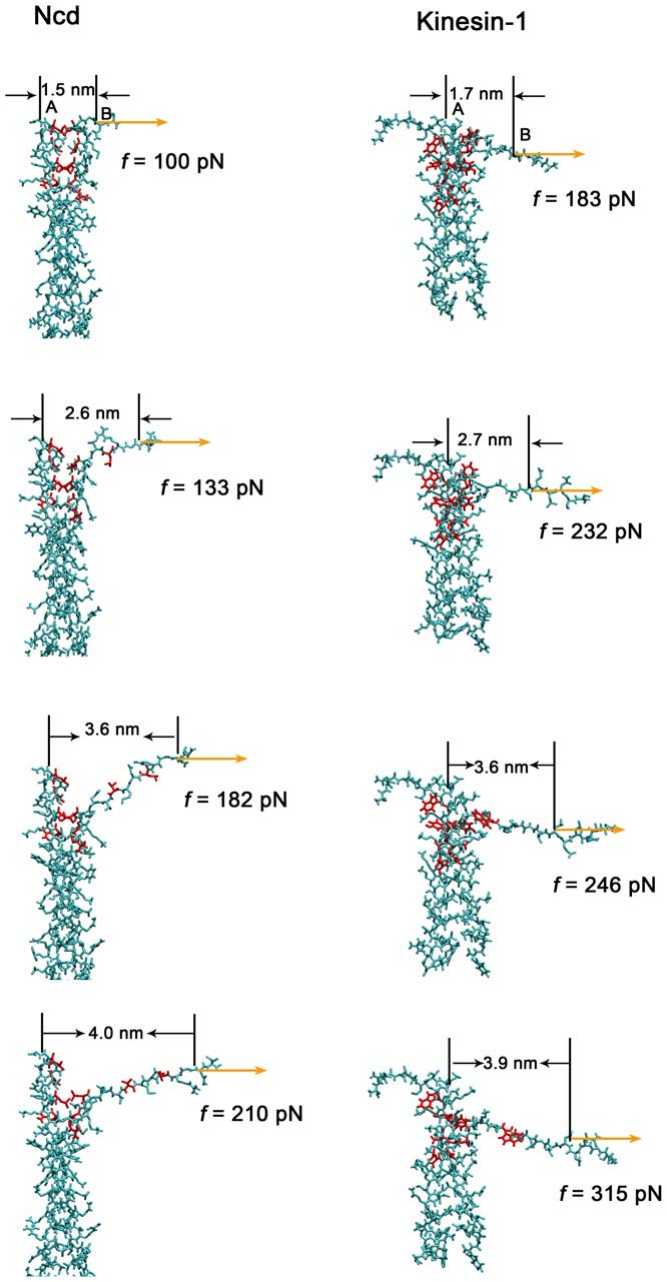
Force induces unwinding of the coiled-coil (Movies S2, S3, S4, S5, S6, S7, S8, S9). Configurations of the coiled-coil (left panel for Ncd and right panel for kinesin-1) by fixing one helix of the coiled-coil and applying constant forces on the residue in the neck linker that is connected to the other helix. The coiled-coil structures of both Ncd and kinesin-1 are maintained under the pulling force smaller than 100 pN for Ncd and the pulling force smaller than 183 pN for kinesin-1. As the pulling forces are further increased, the coiled-coils are gradually unwound and the unwound 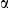-helixes are also unfolded.

In the above, we have simulated the unwinding dynamics of Ncd coiled-coil by applying an external potential on the head domain and a very large change in potential of mean force was obtained to unwind the coiled-coil by only several pairs of residues. To consolidate our results, we now study the unwinding dynamics by directly applying a constant force on the neck linker of Ncd (left panel of Figure 5 and Movies S2, S3, S4, S5) in each simulation. Thus we can search the pulling force that is required to unwind the coiled-coil. In addition, for comparison, we also made similar simulations to the coiled-coil of kinesin-1 (right panel of Figure 5 and Movies S6, S7, S8, S9). Although in inactive state of kinesin-1 the light chain could get close to the head domain and separate the heads and thus may have an impact on the coiled-coil conformation, in active state the light chain keeps a distance from the heads and the coiled-coil [41]. Thus the light chain would have little effect on the conformation of coiled-coil in the active state, under which we studied the stability of coiled-coil.

To avoid the rotational diffusion of coiled-coil which would much increase the size of simulation box, we fixed one helix of the coiled-coil and pulled the neck linker which is connected to the other helix. Actually, this simplification will not obviously change the results of unwinding force when compare with the case by directly pulling both of the helixes without fixing one of them. Here, we defined *r* as the distance between the residue (point A in Figure 5, Asn^348^) in the fixed helix and the residue (point B in Figure 5, Asn^348^), to which the pulling force was applied, along the direction of the force.

**Figure 6:**
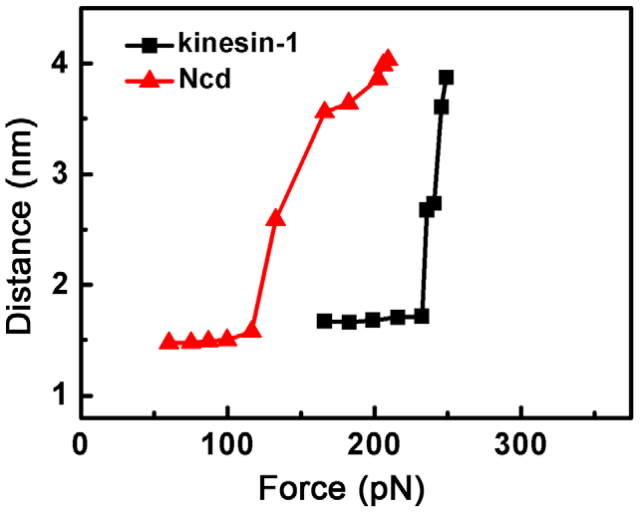
The pulling force *f* versus the distance *r*. The distance *r* is defined as one between the residue (point A in Figure 5) in the fixed helix and the residue (point B in Figure 5), to which *f* is applied, along the direction of *f*. The unwinding force of the coiled-coil for Ncd is about 116 pN and that for kinsin-1 is about 245 pN.

First, we considered the case of Ncd (left panel of Figure 5 and Movies S2, S3, S4, S5). We found that no pair of residues in the coiled-coil of Ncd was unwound when the external force was below a critical value of *f*_uw_ that was larger than 100 pN. Once the force became larger than *f*_uw_, the unwinding of the coiled-coil was initiated. More interestingly, it was seen that, accompanying the coiled-coil unwinding, the unwound 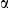-helix was also unfolded (see Figure S3 and Movies S2, S3, S4, S5 for clarity), as noted before when the heads were also included (Figure 1). However, it was noted here that the position on which the external force was applied was shifted from the head domain to the neck linker. Thus it was indicated that the choice of the position on which the force was applied did not affect the unwinding dynamics of the coiled-coil. In addition, the results implied that the unfolding of the 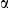-helix is coupled with the unwinding of the coiled-coil, as indicated above in the RMSDs (Figure 2), or the large force resulted in the unfolding of the helix, which in turn induces the coiled-coil unstable or unwinding. To explore the possibility, we simulated the unfolding of the single 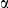-helix of the coiled-coil by applying an external force. The simulation results showed that the unfolding force was smaller than 90 pN (see Text S1, Figure S2 and Movies S10, S11, S12), which is smaller than *f*_uw_ (see also Figure 5), indicating that it is harder to unwind the coiled-coil than to unfold a single 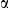-helix. This implied that the coiled-coil conformation could be maintained under the force ranging from 90 pN to 100 pN, which was large enough for the single 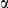-helix to unfold during the 10-ns-long simulation time. Thus it is more possible that the unwinding of coiled-coil is coupled with the unfolding of its 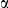-helixes and stability of the helix was enhanced by forming the coiled-coil conformation. To further study the stability of coiled-coil conformation in dimeric Ncd, we also simulated the unwinding of DNA duplex by applying external forces. The unwinding force of DNA duplex was determined to be less than 90 pN (Text S2, Figure S4 and Movies S16, S17, S18), which was smaller than the unwinding force of the coiled-coil, implying that coiled-coil of Ncd was more stable than DNA duplex. The relation of the force for unwinding of the coiled-coil of Ncd versus distance *r* at the end of each simulation is shown in Figure 6 (red line), where it is seen that the critical value *f_uw_* = 116 pN. It is also noted that, in the range of *f* = 116 pN through *f* = 210 pN, the coiled-coil is only partially unwound until the distance *r* reaches *r_max_* (see also Figures 5 and S3).

It is known that the force needed to rupture a bond increases as the pulling speed or the loading rate increases [42]. In our simulations, the loading rate was much higher than that has been used in the available experiments. Thus, the unwinding force of coiled-coils, the unwinding force of DNA duplex and the unfolding force of the single 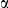-helix in our simulations should be much larger than those obtained in the experiments. Nevertheless, our simulations clearly showed that the coiled-coil of Ncd was more stable than single 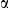-helix or DNA duplex when external forces were stretching them. As it has been experimentally shown, the unfolding force of a single 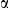-helix was about 25–35 pN under loading rates between 560 and 1600 pNs^−1^ (with the pulling speed being as low as 80 nm s^−1^) [43], [44]; the unwinding force of DNA duplex by using micro-needle is in the range of 10–15 pN under a loading rate smaller than 1 pNs^−1^ [45]. By contrast, the optical trap experiment demonstrated that the unbinding force of the kinesin head from microtubule was less than 8 pN under a loading rate of about 5 pNs^−1^ [46]. Thus, based on our simulations and above experimental results, it was deduced that the unwinding force of Ncd coiled-coil is larger than the unbinding force of kinesin motor head from microtubule.

Then, we considered the case of kinesin-1 (right panel of Figure 5, Movies S6, S7, S8, S9 and black line of Figure 6). It was noted that, similar to the case of Ncd, no pair of residues in the coiled-coil was unwound when the force was below the critical value of *f*_uw_ = 245 pN. In the range of *f* = 245 pN through *f* = 255 pN, the coiled-coil was only partially unwound until the distance *r* reaches *r*_max_. It was also interestingly seen that, accompanying the coiled-coil unwinding, the unwound 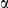 **-**helix was also unfolded (see Figure S3 for clarity). This is consistent with the experimental data on unwinding of a section of coiled-coil belonged to kinesin-1, which indicated that, accompanying the unwinding of the coiled-coil, the 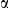-helixes in the coiled-coil were also unfolded [47]. As in the case of Ncd, the force required to unwind the coiled-coil of kinesin-1 is also larger than that to unfold the single 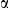-helix that forms the coiled-coil (see Text S1, Figure S2 and Movies S13, S14, S15). The results implied again that the unwinding of coiled-coil is coupled with the unfolding of its helixes and the stability of the helix was enhanced by forming the coiled-coil conformation.

Comparing the case of Ncd (red line of Figure 6) with that of kinesin-1 (black line of Figure 6), it was seen that the critical value of *f*_uw_ for the case of kinesin-1 is larger than that of Ncd and, thus, the coiled-coil of kinesin-1 is more stable than that of Ncd. As a result, we concluded that no such conformation can occur that the coiled-coil is unwound when both heads of kinesin-1 are binding to microtubule. This is consistent with the experimental data, showing that the function of kinesin-1 was little affected when its coiled-coil was prevented to unwind by disulfide cross-linking [48].

### Analyses of coiled-coil stability based on their sequences

**Figure 7:**
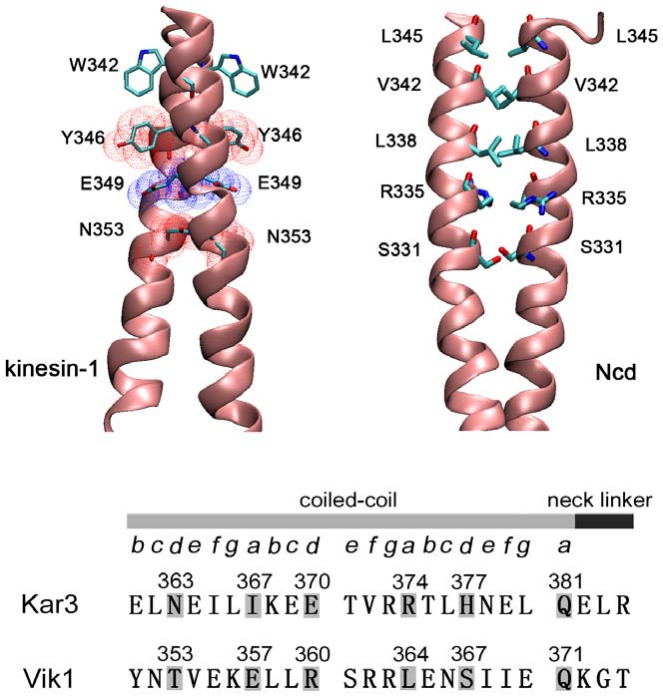
Configurations and sequences of the coiled-coil. (Upper panel) The coiled-coil segments of kinesin-1 (left) and Ncd (right). (Upper left) Two indole rings of residues W342 are projected out from the hydrophobic core, with each indole ring extending to the opposite helix. E349 has negatively charged side chains (blue) which is sandwiched by the side chains of Y346 and N353 which have positively charged side chains (red). (Upper right) The three pairs of residues (L345, V342, and L338) in the top of the coiled-coil segment have hydrophobic side chains while the two pairs of residues (R335 and S331) in the bottom have charged or polar side chains. (Lower panel) Sequences of the segment that is composed of the neck linker and a fraction of the coiled-coil of Kar3 and Vik1. The neck linker of Kar3 is assumed to be composed of 3 residues E382, L383, and R384. The polar residues N363, H377 and Q381 in Kar3 could interact with the polar residues T353, S367 and Q371 in Vik1, respectively, by forming hydrogen bonds. The charged residues, E370 and R374 in Kar3, E357 and R360 in Vik1, could form a conformation to avoid the repulsive part of the electronic interaction, similar to that in the coiled-coil of kinesin-1. The two types of the interaction could greatly enhance the stability of the coiled-coil in heterodimeric Kar3/Vik1.

The difference in the critical value of *f*_uw_ between the case of kinesin-1 and that of Ncd could result from the difference in properties of the helixes which form the coiled-coil. Generally, when two 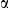-helixes form a coiled-coil, the inter-helix interaction is mainly attributed to the attractive interaction of the side chain in the helix residues which have a heptad repeat sequence (*abcdefg*)*_n_*. The residues at *a* and *d* sites of the helix sequence are usually constituted by hydrophobic side chain residues [49]. If two such helixes become close to each other, the attractive interaction between the helixes induces the formation of hydrophobic cores, thus forming the coiled-coil structure. For the coiled-coil of kinesin-1, the sequence components does not rigorously obey the rule of hydrophobic residues at *a* and *d* sites. However, two distinct properties are very important for the stability of its coiled-coil structure. One is the presence of the charged residue (Glu^349^) and polar residues (Tyr^346^ and Asn^353^) at the end of coiled-coil near the head (Figure 7, upper left). These side chain residues could interact with other polar side chains in the opposite helix by a subtle position arrangement so that the repulsive component of the interaction is avoided [50]. The other property is that the indole ring of Trp^342^ is exposed to the solvent. The two indole rings in the two helixes extend out from the hydrophobic core positions, with each indole ring extending to the opposite helix, thus forming a pair of “arms" that “hug" the coiled-coil (Figure 7, upper left). These rings are thought to greatly enhance the stability of the coiled-coil structure [50], [51]. By contrast with kinesin-1, the coiled-coil of Ncd has more regular hydrophobic amino acids at *a* and *d* sites and has no residues with ring side chain in the helix. However, the side chains of the two residues Arg^335^ are charged and extend out from the hydrophobic core positions by their exclusive interaction (Figure 7, upper right), which makes the coiled-coil of Ncd not as stable as that of kinesin-1 or even makes the coiled-coil unable to form when a single mutant residue is introduced in the neck region [52]. Similarly, the very narrow force range for unwinding of the coiled-coil of kinesin-1 could also be due to both the effect of the indole rings and the electrostatic attractive interaction in the coiled-coil segment adjacent to the neck linkers, as just mentioned above.

The crystal structure of Kar3 showed that its head domain starts at Gly^385^ [37]. If we assumed that the neck linker of Kar3 has 3 residues (from Glu^382^ to Arg^384^), from the analysis of the sequence of the neck region of Kar3 and that of Vik1 (Figure 7, bottom) it was indicated that the residues in the neck region of Kar3 could interact with that of Vik1 to form a stable coiled-coil, which was described as follows. Both Kar3 and Vik1 contain the same residue Gln at *a* site (Q381 in Kar3 and Q371 in Vik1) (Figure 7, bottom). Gln is a polar residue which has amino group (-NH_2_) and oxo group ( = O) at the end of its side chain. These components could form two hydrogen bonds, implying that Gln^381^ in Kar3 could form two hydrogen bonds with Gln^371^ in Vik1. Similarly, two pairs of amino acids (His^377^ in Kar3 with Ser^367^ in Vik1 and Asn^363^ in Kar3 with Thr^353^ in Vik1) in the neck region of Kar3/Vik1 could form hydrogen bonds. These hydrogen bonds could greatly enhance the stability of the coiled-coil. In addition, positively charged residues (Arg^374^ in Kar3 and Arg^360^ in Vik1) and negatively charged residues (Glu^370^ in Kar3 and Glu^357^ in Vik1) could be arranged in positions so that the repulsive interaction of those charge groups is avoided and only the attractive interaction is left, which is similar to the case of kinesin-1. The above two types of the interaction generally make the coiled-coil more stable than the solely hydrophobic interaction by which the coiled-coil of Ncd is formed stably. As a result, the coiled-coil of heterodimer Kar3/Vik1 is most probable to be more stable than that of Ncd and, at least, to be as stable as that of Ncd. Thus, it was deduced that the coiled-coil of Kar3/Vik1, like Ncd, is also impossible to unwind during the dimer walking on microtubule.

## Discussion

Since the neck linkers of kinesin Ncd are very short, only by unwinding the coiled-coil region can its two heads be separated by a large distance so that they can simultaneously bind the two successive binding sites along one protofilament of the microtubule. As a result, in rigor state of Ncd, only one head can bind to the microtubule [22], [23], [24], [25]. By contrast, kinesin-1 has two long flexible neck linkers. By docking the neck linker of the trailing head, undocking the neck linker of the leading head and stretching the undocked neck linker, the two heads can be easily separated to make them simultaneously bind the two successive binding sites along one protofilament of the microtubule [15], [53], [54], [55]. Thus, in rigor state, the two heads are bound to the microtubule for kinesin-1.

**Figure 8:**
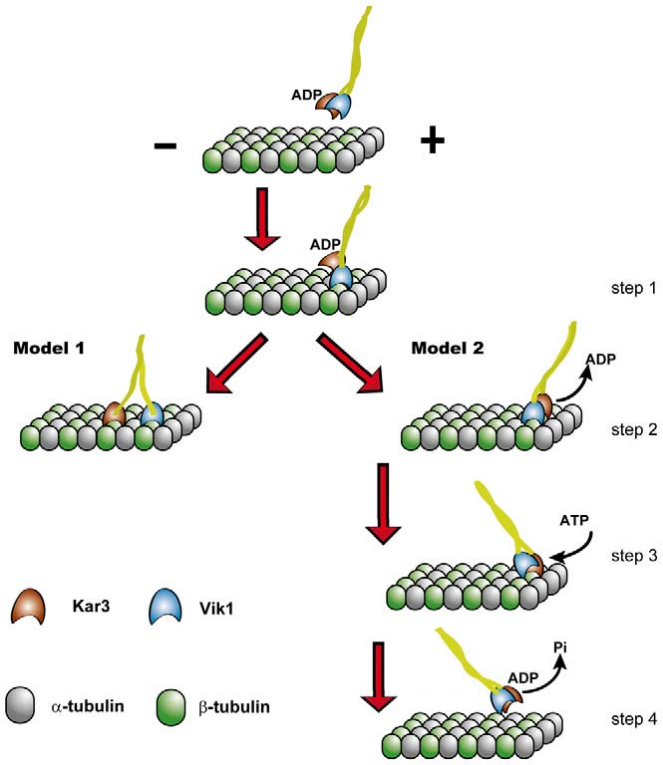
The two models for Kar3/Vik1 heterodimer walking on microtubule. In model 1, the two heads of the dimer bind to the same microtubule protofilament via coiled-coil unwinding. Our present results indicated that this model is impossible. In model 2, the two heads bind to two adjacent microtubule protofilaments without coiled-coil unwinding (step 2). Our present results supported this model. In model 2, after an ATP binds to Kar3, the rotation of its neck domains drives, via the coiled-coil, Vik1 detaching from the microtubule, making the cargo move toward the minus end (step 3). After ATP hydrolysis and then the release of Pi, Kar3 becomes bound weakly to the microtubule and then, due to the thermal noise, it detaches from the microtubule (step 4).

Like Ncd, Vik1 of Kar3/Vik1 also has a very short neck linker (with only 3 residues). In addition, based on the sequence analysis of Kar3 and the comparison of it with that of Vik1, we infer that Kar3 also has a short neck linker (with only 3 residues). Thus, like homodimeric Ncd, without the unwinding of its coiled-coil, the two heads of heterodimeric Kar3/Vik1 is unable to simultaneously bind the two successive binding sites along one protofilament of the microtubule. As a result, a direct expectation would be that, in rigor state, only one head, Kar3, of Kar3/Vik1 can bind to the microtubule, just like Ncd, because not only both Kar3/Vik1 and Ncd belong to the same kinesin subfamily but also Vik1 lacks nucleotide binding site and only Kar3 can catalyze the hydrolysis of ATP. However, experiment data have indicated that both heads of Kar3/Vik1 can bind to microtubule in ADP-bound state [36]. Thus, two different models have been proposed for the rigor state of Kar3/Vik1 during its walking along microtubule (Figure 8). The first model proposes that its coiled-coil can be unwound, thus allowing the two heads to simultaneously bind the two successive binding sites along one protofilament of the microtubule, analogous to the case of kinesin-1 (Figure 8, model 1) [36], [40]. As our present results indicated, this model is most impossible. In the second model, the coiled-coil of Kar3/Vik1 is not necessary to unwind and the two heads bind to the successive two binding sites on the adjacent protofilaments of microtubule. In this rigor state without unwinding of the coiled-coil, the two heads can be separated by the distance (about 4 nm) between the two successive binding sites on the adjacent protofilaments (Figure 8, step 2 in model 2). Our present results supported this model.

The structure of Ncd showed that its neck linker is connected to the 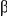 sheet that is buried into the core of its head domain, which is different from the case of kinesin-1, whose structure showed that its neck linker can form 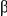 sheets which are docked on the surface of head domain. Like Ncd, the structure of Kar3 also showed that the neck linker should be connected to the 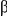 sheet that is buried into the core of Kar3. This similarity of Kar3 to Ncd makes Kar3 most possible to use the similar mechanism to Ncd to walk on the microtubule, i.e., by rotating the neck domain around the head for some degree to generate force in coiled-coil region [28]. Based on this argument, the second model is completed as follows. First, Vik1 binds to the binding site on one protofilament (step 1). Then ADP-bound Kar3 binds weakly to the binding site on the adjacent protofilament and, activated by the microtubule, ADP is released, thus Kar3 becoming bound strongly to the microtubule (step 2). In this rigor state, an ATP binds to Kar3 and the ensuing rotation of its neck domains drives, via the coiled-coil, Vik1 detaching from the microtubule, making the cargo move toward the minus end of microtubule (step 3). After ATP hydrolysis and then the release of Pi, Kar3 becomes bound weakly to the microtubule and then, due to the thermal noise, it detaches from the microtubule (step 4). Thus, a mechanochemical coupling cycle is completed.

In addition, it is interesting to note here that, in some dimeric myosin motors such as myosin V and myosin VI, the two head domains are also connected by a rigid coiled-coil [4]. Thus, the studies in the present work would also have an implication in understanding the mechanism of those dimeric myosin motors moving along actin. For example, a model proposed that the walking of myosin VI along actin requires the unwinding of its coiled-coil [56], while another model suggested that the myosin VI runs along actin and thus the coiled-coil is unnecessary to unwind [57].

In summary, our molecular dynamics simulations showed that both dimeric Ncd and kinesin-1 have very stable coiled-coils. It is required a large force or a large change in potential of mean force to unwind their coiled-coils by only several pairs of residues, with the force being higher than the unbinding force of the kinesin head from the microtubule. Based on these results and the comparison of the sequence between the coiled-coil of heterodimeric Kar3/Vik1 and those of the homodimeric Ncd and kinesin-1, it was deduced that the coiled-coil of the Kar3/Vik1 would also be very stable. Thus, we concluded that, for Ncd and kinesin-1, their coiled-coils are not unwound while, for Kar3/Vik1, its coiled-coil is most probable not to be unwound during their walking along the microtubule.

## Materials and Methods

We used the structures of truncated dimeric Ncd (PDB 1CZ7) [26] and kinesin-1 (PDB 2KIN) [58] from the RCSB protein data bank. We chose these two structures because they are the only dimeric crystal structures available now. The missing atoms in the neck domain of 1CZ7 were added by using the software Swiss-PdbViewer3.7. Then a 100-piconseconds molecular dynamics simulation was carried out to make the molecular conformation in nearly thermodynamic equilibrium. The molecular dynamics simulation was carried out by using GROMACS4 on a cluster which contains 3 nodes. Each node was constituted by 8 Intel Xeon CPUs. The AMBER force field was used. The temperature was set to be 310 K and the step size was set as 2 fs. A water/peptide system was constructed using the spc216 flexible water model and a minimum peptide-to-edge distance of 1 nm and periodic boundary conditions were used to limit edge effects. Counter-ions were added to neutralize the charge of the system. The above simulation parameters were also used in all the following simulations. For each simulation, a preliminary simulation of 500 ps was run by incorporating with both Berendsen temperature-coupling and Parrinello-Rahman pressure-coupling schemes.

For generating the unwinding conformation of coiled-coils we first focus on the nucleotide-free state of Ncd. The ADP molecules in 1CZ7 were removed. One head of the Ncd dimer was fixed with position restraints algorithm in GROMACS4. Then a series of simulations were carried out with a harmonic potential applied on the heads to pull away the other head (see Figure 1) by a distance of about 2 nm, as did in the pulling force experiments. The umbrella pulling option in the pull code of GROMACS4 was used to generate the harmonic potential and the force constant was set to be 1000 kJmol^−1^nm^−2^. The pulling direction was along the line which connects the centers of mass of the heads. To avoid the affect of pulling speed or pulling rate on the final conformation where the coiled-coil was unwound, the simulations were run at different pulling speeds. The pulling speeds are ranged from 2
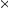
10^−2^ nm/ps to 2
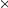
10^−4^ nm/ps. For each pulling speed, 5 independent simulations were run and then all the final conformations in these simulations were compared. To consider the effect of the form of the external force field, a linear potential or constant force was applied by GROMACS4 instead of the harmonic potential and the same procedure was carried out. The linear potential or the constant force was also generated by constant force option in the pull code of GROMACS4. The force constant here was set to be 500 kJmol^−1^nm^−1^. To test the effect of the nucleotide states, the ADP molecules in structure 1CZ7 were conserved, the same harmonic potential was applied and the simulations were run at the pulling speed of 2
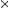
10^−3^ nm/ps. Five independent simulations were run again. The topology files of ADP molecule were generated by the online PRODRG server. In above calculations the simulation box was with a volume of 13.5 nm
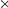
8.7 nm
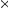
13.8 nm, with 13.8 nm along the force direction and 13.5 nm along the coiled-coil axis, and 49104 water molecules were present in the box. It totally took 250 ns simulation time for all the 55 conformational searching simulations.

From a series of molecular configurations of dimeric Ncd which was in nucleotide-free state during the pulling process under a pulling rate of 2
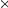
10^−4^ nm/ps (Figure 1), we calculated the potential of mean force resulting from the conformational change of the coiled-coil. The potential of mean force was calculated by using Weighted Histogram Analysis Umbrella Sampling Method [59], [60]. We used only the configurations of the coiled-coil region by cutting off the heads and fixing one helix of the coiled-coil by position restraints. The harmonic potential which was introduced by the umbrella sampling method was imposed to the molecule in each configuration with a force constant of 1000 kJmol^−1^nm^−2^, as used before. There are total 11 simulation windows and the pacing of the simulation windows was less than 0.2 nm so that the histograms of the configurations could overlap with their neighbor windows. The simulation time for each window lasted 10 ns. The large force constant and simulation time scale ensured that values of 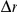 distribute around the sampling points in a Gaussian form.

We then used another way to search the force that is required to unwind the coiled-coil. The two heads of both Ncd and Kinesin-1 were cut off from the dimers and we then fixed one neck helix by position restraints algorithm and exerted a constant force on the neck linker that is connected to the unrestrained neck helix. The direction of the constant force was still along the line which passes through the centers of mass of the two heads. A series of simulations were performed under different constant forces to estimate the unwinding force of the coiled-coils. For each constant force 3 independent simulations were run and each simulation lasted 10 ns. 60 simulations have been run in the unwinding force searching and it totally took 600 ns of simulation time. The simulations were with a box volume of 8 nm
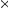
4 nm
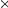
12 nm, with 8 nm along the force direction and 12 nm along the helix axis. Thus, the maximal displacement of the pulling groups on which the pulling force was applied could reach 4 nm, which was sufficient to study the unwinding of the coiled-coil. The simulation box contained 10242 water molecules in the case of Ncd and 9224 water molecules in the case of kinesin-1. We also applied constant forces to single 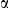-helixes and DNA duplex in the same simulation conditions to evaluate their unfolding and unwinding forces, respectively (see Text S1 and Text S2). These forces were compared with the unwinding forces of coiled-coils to study the coiled-coil stability.

## Supporting Information

Text S1
**Unfolding** 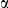 **-helixes under external forces.**
(DOC)

Text S2 **Unwinding of DNA duplex under external forces.**
(DOC)

Figure S1 **Snapshots of the pulling trajectories of Ncd dimer under linear external potential and ADP bound state.** (Upper) The start and end configurations of Ncd dimer under linear external potential without ADP bound. (Bottom) The start and end configurations of Ncd dimer under harmonic external potential with ADP bound.
(TIF)

Figure S2
**Unfolding of the** 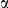 **-helixes by external force (Movies S10, S11, S12, S13, S14, S15).** (Left) Unfolding of the helix that forms the coiled-coil of Ncd. The force required for unfolding of the helix lies between 80 pN and 90 pN. (Right) Unfolding of the helix that forms the coiled-coil of kinesin-1. The force required for unfolding of the helix is about 140 pN.(TIF)

Figure S3 **Force induces unwinding of the coiled-coil.** Configurations of the coiled-coil in ribbon format (left panel for Ncd and right panel for kinesin-1) by fixing one helix of the coiled-coil and applying constant forces on the residue in the neck linker that is connected to the other helix.
(TIF)

Figure S4 **Unwinding of DNA duplex by external force (Movies S16, S17, S18).** An external force was applied to the phosphorus atom near the 3*′* end of one strand and another external force of the same magnitude but in opposite direction was applied to the phosphorus atoms near the 5*′* end of another strand. The force required to unwind the DNA duplex lies between 80 pN and 90 pN.
(TIF)

Movie S1
**Conformational Trajectories for Ncd dimer under the external potential, with the pulling speed of 2** 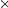 **10^−4^ nm/ps.** For clarity, the area of the box shown here is smaller than that used in the simulation.(AVI)

Movie S2 **Unwinding of Ncd coiled-coil under an external force of 100 pN.** For clarity, the area of the box shown here is smaller than that used in the simulation.
(AVI)

Movie S3 **Unwinding of Ncd coiled-coil under an external force of 133 pN.** For clarity, the area of the box shown here is smaller than that used in the simulation.
(AVI)

Movie S4 **Unwinding of Ncd coiled-coil under an external force of 182 pN.** For clarity, the area of the box shown here is smaller than that used in the simulation.
(AVI)

Movie S5 **Unwinding of Ncd coiled-coil under an external force of 210 pN.** For clarity, the area of the box shown here is smaller than that used in the simulation.
(AVI)

Movie S6 **Unwinding of kinesin-1 coiled-coil under an external force of 183 pN.** For clarity, the area of the box shown here is smaller than that used in the simulation.
(AVI)

Movie S7 **Unwinding of kinesin-1 coiled-coil under an external force of 232 pN.** For clarity, the area of the box shown here is smaller than that in the simulation.
(AVI)

Movie S8 **Unwinding of kinesin-1 coiled-coil under an external force of 246 pN.** For clarity, the area of the box shown here is smaller than that used in the simulation.
(AVI)

Movie S9 **Unwinding of kinesin-1 coiled-coil under an external force of 315 pN.** For clarity, the area of the box shown here is smaller than that used in the simulation.
(AVI)

Movie S10
**Unfolding of the** 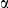 **-helix that forms Ncd coiled-coil under an external force of 80 pN.** For clarity, the area of the box shown here is smaller than that used in the simulation.(AVI)

Movie S11
**Unfolding of the** 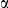 **-helix that forms Ncd coiled-coil under an external force of 90 pN.** For clarity, the area of the box shown here is smaller than that used in the simulation.(AVI)

Movie S12
**Unfolding of the** 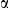 **-helix that forms Ncd coiled-coil under an external force of 100 pN.** For clarity, the area of the box shown here is smaller than that used in the simulation.(AVI)

Movie S13
**Unfolding of the** 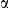 **-helix that forms kinesin-1 coiled-coil under an external force of 130 pN.** For clarity, the area of the box shown here is smaller than that used in the simulation.(AVI)

Movie S14
**Unfolding of the** 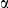 **-helix that forms kinesin-1 coiled-coil under an external force of 140 pN.** For clarity, the area of the box shown here is smaller than that used in the simulation.(AVI)

Movie S15
**Unfolding of the** 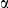 **-helix that forms kinesin-1 coiled-coil under an external force of 150 pN.** For clarity, the area of the box shown here is smaller than that used in the simulation.(AVI)

Movie S16 **Unwinding of DNA duplex under an external force of 80 pN.** For clarity, the area of the box shown here is smaller than that used in the simulation.
(AVI)

Movie S17 **Unwinding of DNA duplex under an external force of 90 pN.** For clarity, the area of the box shown here is smaller than that used in the simulation.
(AVI)

Movie S18 **Unwinding of DNA duplex under an external force of 100 pN.** For clarity, the area of the box shown here is smaller than that used in the simulation.
(AVI)
