# Supplementary material for: Are Coiled-Coils of Dimeric Kinesins Unwound during Their Walking on Microtubule?
Source: PLoS One. 2012 Apr 27;7(4):e36071. doi: 10.1371/journal.pone.0036071 (PMC3338639; doi:10.1371/journal.pone.0036071)
Supplement: Text S1 — Unfolding -helixes under external forces. (DOC) [file pone.0036071.s001.doc]

**Text S1. Unfolding α-helixes under external forces**

The structural data of the α-helixes which form the coiled-coil of Ncd and kinesin-1 were taken from PDB files 1CZ7 and 2KIN, respectively. In our simulations, we considered the helixes containing only 30 residues which are close to the neck linkers. We fixed one end of the helix and applied a constant pulling force on the residue at the other end, to which the neck linker is connected in the original structure. The force is along the direction which is parallel with the helical axes. For Ncd, the simulation box was 15 nm$\times$3.8 nm$\times$3.6 nm, with 15 nm along the helical axis, and 6387 water molecules were present in the box. For kinesin-1, the box was 3.6 nm$\times$15 nm$\times$3.6 nm, with 15 nm along the helical axis, and there were 6166 water molecules. Both the systems were neutralized with Na+ ions. The temperature was set to 310K and each simulation lasted for 10 ns.

First, we considered the case of Ncd (left panel of Figure S2 and Movies S10 – S12). We found that the force required for unfolding of the helix that forms the coiled-coil of Ncd is in the range between 80 pN and 90 pN. The results for the case of kinesin-1 are shown in the right panel of Figure S2 and Movies S13 – S15. It is seen that the force required for unfolding of the helix that forms the coiled-coil of kinesin-1 is about 140 pN.
