# Supplementary material for: Are Coiled-Coils of Dimeric Kinesins Unwound during Their Walking on Microtubule?
Source: PLoS One. 2012 Apr 27;7(4):e36071. doi: 10.1371/journal.pone.0036071 (PMC3338639; doi:10.1371/journal.pone.0036071)
Supplement: Text S2 — Unwinding of DNA duplex under external forces. (DOC) [file pone.0036071.s002.doc]

**Text S2. Unwinding of double strands DNA under external forces**

A sequence of 15 base-pairs (5’-AAGCTGGAATTTGCT-3’) was randomly chosen. The conformation of this dsDNA molecule was generated by software Hyperchem7.5. After the conformation was got we used GROMACS4 to run the free MD simulation for 5 ns and obtained an equilibrium structure. Then lower half of the dsDNA molecule in Figure S4 was fixed, and a pair of external forces was applied to the first pair of phosphorus atoms at the end of the upper half of the dsDNA. The forces were vertical to DNA axis and parallel with the line which passed through the two phosphorus atoms. The simulation box was 8 nm$\times$7 nm$\times$4.2 nm, with 7 nm along DNA axis and 8 nm along the force direction. 7425 water molecules were added in the box together with 28 Na+ ions for neutralization. The temperature was set to 310K and each simulation lasted for 10 ns.

The results of the simulations showed that the DNA were unwound by about 4-5 base pairs when the force was 100 pN, 3-4 base pairs when the force was 90 pN and none of the base pairs was opened when the force was 80 pN (Figure S4 and Movies S16-S18). Since the lower half of the DNA was fixed, the molecule was not allowed to rotate so that the further unwinding was prevented when the external force induced the collision of base pairs.
