# Supplementary figures and images for: Are Coiled-Coils of Dimeric Kinesins Unwound during Their Walking on Microtubule?
Source: PLoS One. 2012 Apr 27;7(4):e36071. doi: 10.1371/journal.pone.0036071 (PMC3338639; doi:10.1371/journal.pone.0036071)

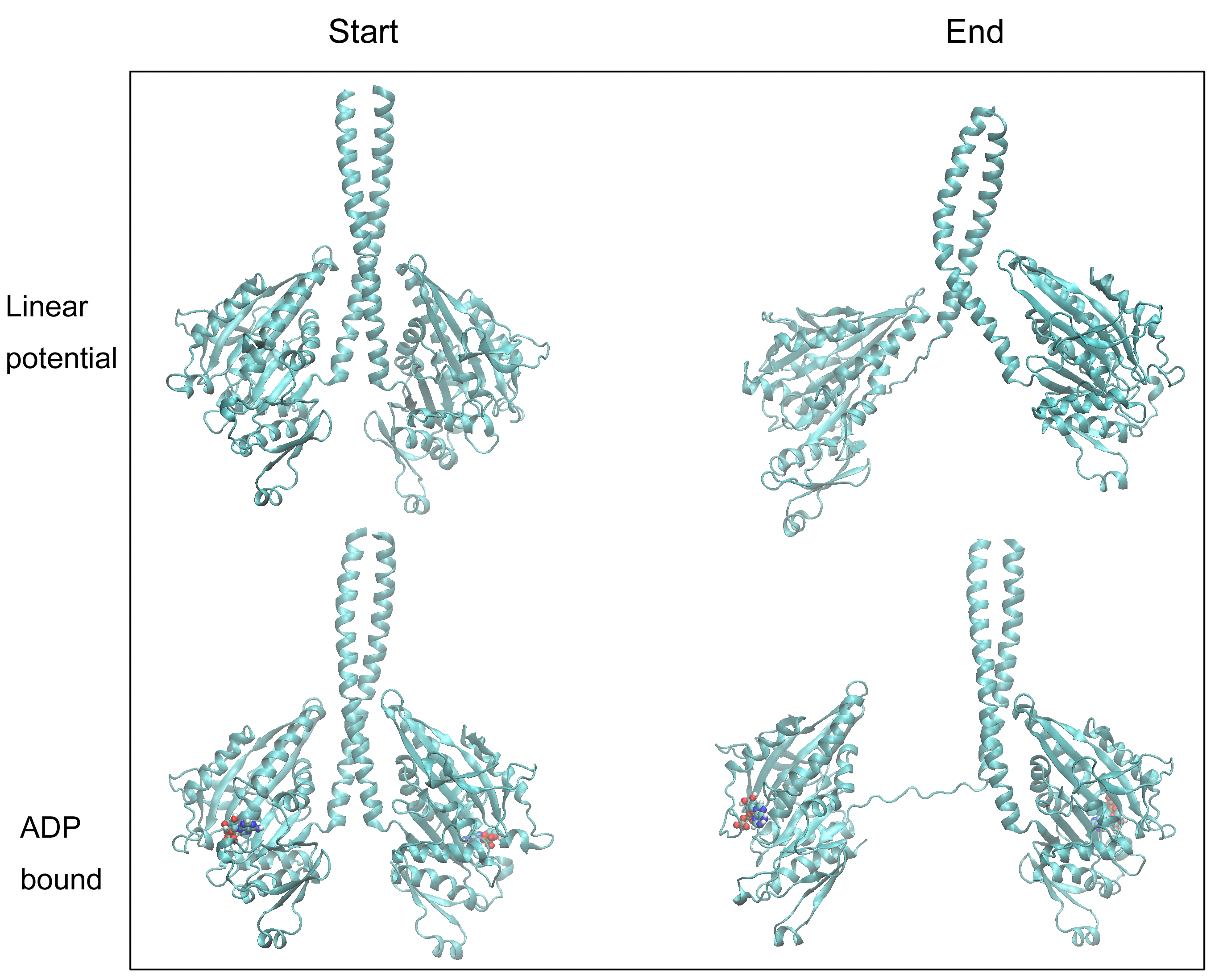

Supplement: Figure S1 — Snapshots of the pulling trajectories of Ncd dimer under linear external potential and ADP bound state. (Upper) The start and end configurations of Ncd dimer under linear external potential without ADP bound. (Bottom) The start and end configurations of Ncd dimer under harmonic external potential with ADP bound. (TIF) [file pone.0036071.s003.tif]

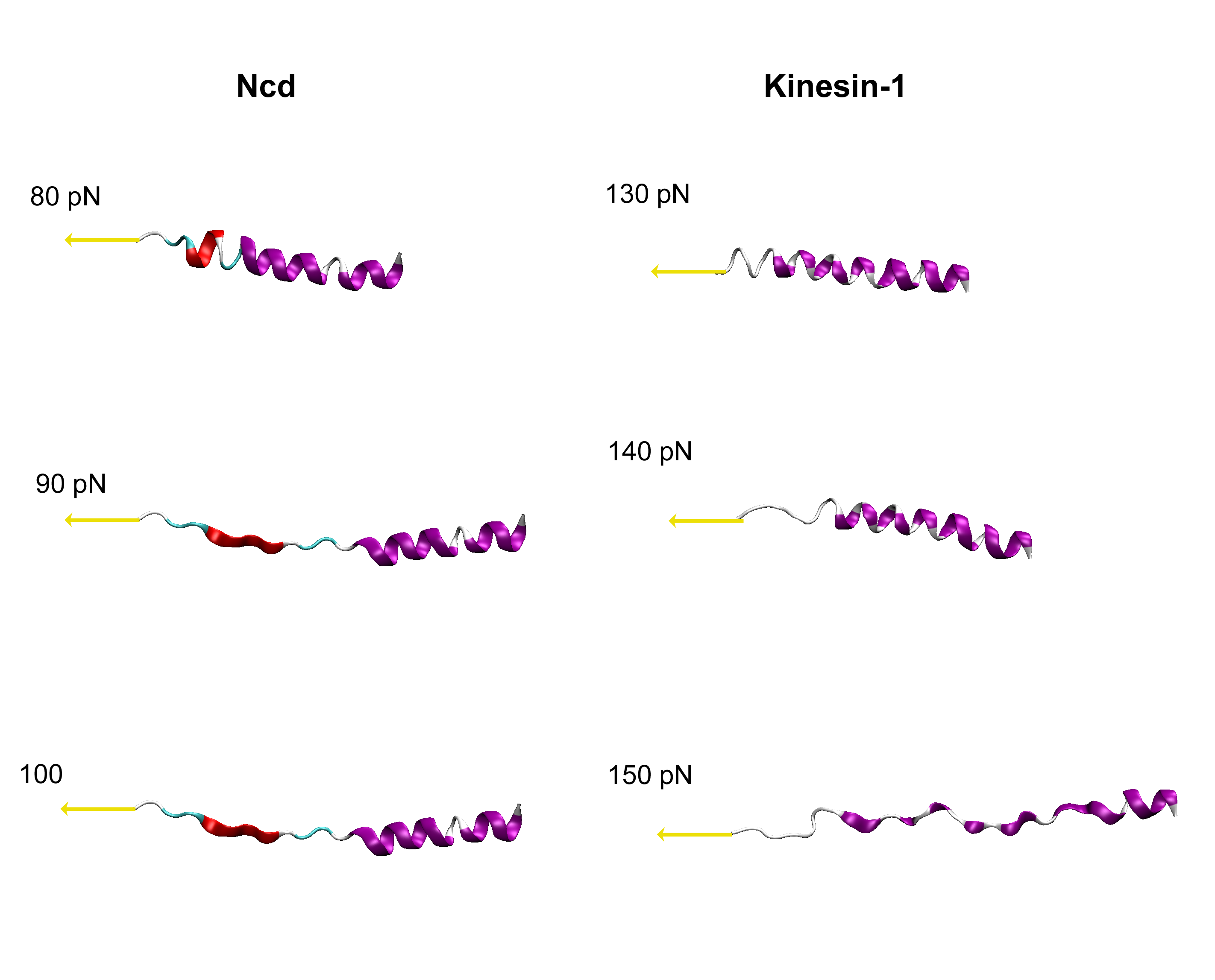

Supplement: Figure S2 — Unfolding of the -helixes by external force (Movies S10, S11, S12, S13, S14, S15). (Left) Unfolding of the helix that forms the coiled-coil of Ncd. The force required for unfolding of the helix lies between 80 pN and 90 pN. (Right) Unfolding of the helix that forms the coiled-coil of kinesin-1. The force required for unfolding of the helix is about 140 pN. (TIF) [file pone.0036071.s004.tif]

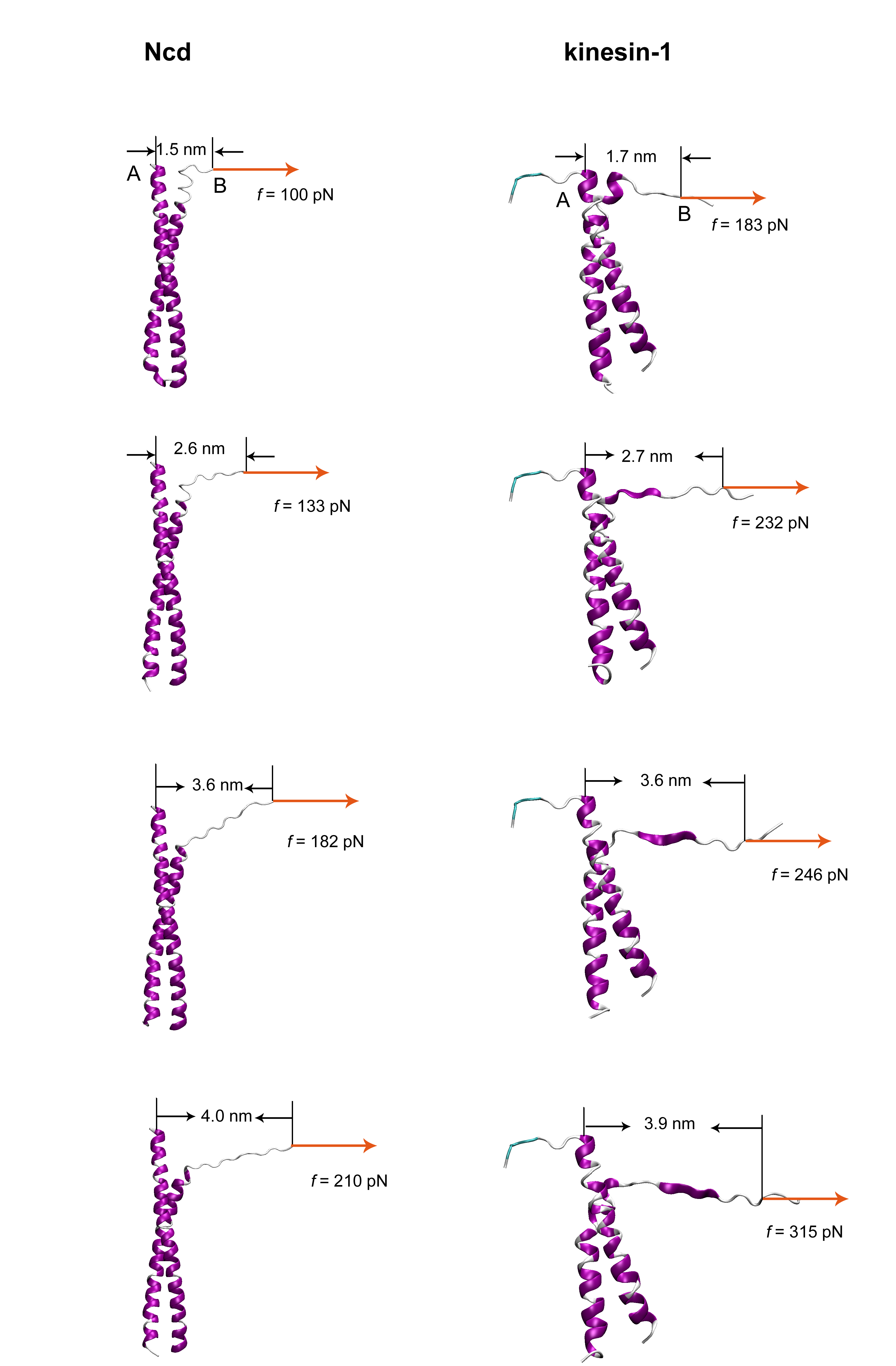

Supplement: Figure S3 — Force induces unwinding of the coiled-coil. Configurations of the coiled-coil in ribbon format (left panel for Ncd and right panel for kinesin-1) by fixing one helix of the coiled-coil and applying constant forces on the residue in the neck linker that is connected to the other helix. (TIF) [file pone.0036071.s005.tif]

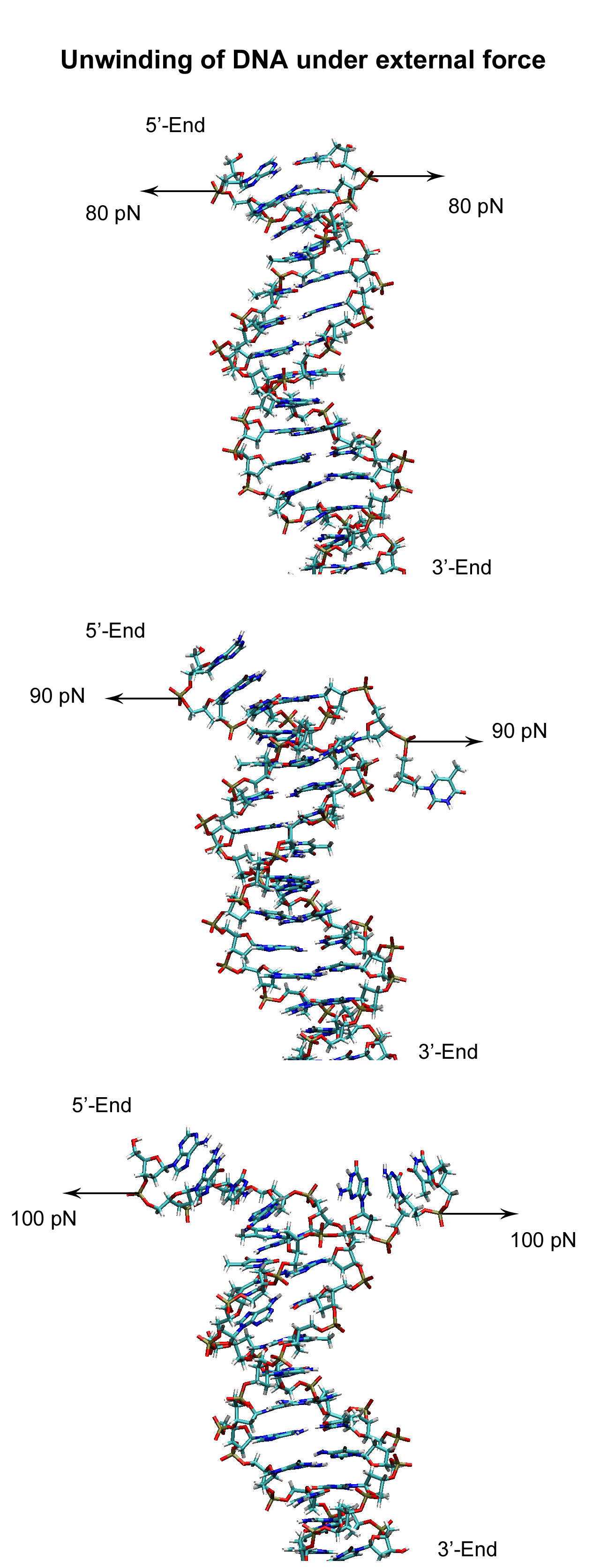

Supplement: Figure S4 — Unwinding of DNA duplex by external force (Movies S16, S17, S18). An external force was applied to the phosphorus atom near the 3′ end of one strand and another external force of the same magnitude but in opposite direction was applied to the phosphorus atoms near the 5′ end of another strand. The force required to unwind the DNA duplex lies between 80 pN and 90 pN. (TIF) [file pone.0036071.s006.tif]
